# Event and Comment

**Published:** 1918-09

**Authors:** 


### DENTAL REGISTER

Vol. LXXII SEPTEMBER, 1918 No. 9

### EVENT AND COMMENT

**The National Meeting** The most important event of the year, perhaps, is the annual gathering of the dentists of the whole country in a formal convention to record the doings of the past year and to lay out a working schedule for the coming year of professional activities. The convention held in Chicago August 5-9, was undoubtedly the largest gathering of dentists ever held. More than six thousand dentists registered. This large attendance is rather remarkable, considering the fact that in these times our people are making extraordinary efforts to economize both in time and money, and also in view of the fact that many dentists had to travel long distances and the weather was extremely hot and uncomfortable. If compared with the attendance at the meeting of the American Medical Association last June in Chicago, when only five thousand members attended, and in view of the facts that there are more than one hundred thousand medical practitioners and less than forty thousand dental practitioners in the country, it seems somewhat remarkable. It indicates, at least, that in spite of the unusual conditions under which we are living that the dental profession is actively interested in its own development and at the same time is doing its part in helping to carry on the war work. It developed in the proceedings at Chicago that not only was our profession doing its part in all war work in a most satisfactory manner professionally, but that nearly twice as many dentists were already enlisted as could be used at the present time. It was also shown that the civil practitioners have contributed voluntarily more than half a million operations to enlisted soldiers. This should not only show that the profession was fully alive to its patriotic duties, but that it was not profligate in these serious times. In fact, it seemed to us that the general feeling prevailed that it was an occasion for getting together in convention to receive mutual encouragement and to further develop concerted and sympathetic cooperation in our war duties as well as in professional attainments. War support was, of course, the prevailing comment in all the exercises, and yet we have never attended a meeting where so strenuous a program of professional matters was carried out. The exercises were begun promptly on the scheduled time of the printed program and there were no vacancies in the program for frivolous entertainments. The officers and local committees could not have done more to make the meeting successful and profitable to everyone. The weather only was faulty and this, of course, no one could foresee or prevent.

It is not possible here to record in even the most general statement the tremendous amount of work done in that one week. In fact, it is difficult to give any good description of a single event that should receive especial mention. We have been attending national conventions for more than thirty years, and we believe the 1918 meeting accomplished more actual worth-while work than any of its predecessors. The published proceedings of the coming year will show in part what was accomplished, but no one who attended the sessions as fully as was possible could have witnessed one-half of the proceedings. In fact, the remark was frequently made that this convention was getting altogether too large for its best usefulness. If it were possible to divide the country into three or four sections for the clinical features only, it might make this part of the program more satisfactory, as it was practically impossible to get into satisfactory distance of any of the clinics. If this convention is to be as large as at present, it will certainly become necessary to adopt some other method of giving clinics, or they might as well be omitted from the program entirely.

One of the most interesting events of the meeting was the dedication of the monument to Dr. G. V. Black. It is a very excellent reproduction in bronze, more than life size, mounted on a gray granite settle. It is located in the south end of Lincoln Park, directly opposite the Plaza Hotel. The place is not far from where Dr. Black lived at one time, and is readily reached by street cars from the business district of Chicago. We wish it could have been located near the new Field Museum in Grant Park, but this was not practicable, as this park at this time is in process of construction. Some day Chicago will have a dental museum and possibly a dental research laboratory and it would be a grand thing if it could be near the Field Museum so that it would be most readily visited by out-of-the-city dentists.

Chicago is a great city and her dentists are probably more progressive and have more professional pride in professional enterprises than any other city in the country. They certainly know how to make professional gatherings interesting. It can only be attributed to their training of many years; their spirit of professional devotion; fraternal cooperation, and above all, to their unbounded generosity.

### BLACK HISTORICAL EXHIBIT

On the occasion of the dedication of the monument to Dr. Black, it seemed appropriate that there should be exhibited some of the material things which will serve to remind the older and inspire the younger practitioners with the work of dentistry's greatest scientific investigator, writer and teacher. The officers of the National Dental Association, therefore, requested Dr. William Bebb to prepare a Black Historical Exhibit, and arranged for its display in the South Parlor of the Auditorium Hotel.

There never has been shown at a dental meeting a historical collection of such magnitude and interest. It covers the entire period of Dr. Black's life from his entry into the practice of dentistry in 1858 up to the time of his death in 1915. It not only brings one into touch with his dental activities, but presents many phases of his work in other lines and of his home life not heretofore recorded.

This exhibit consists of a reproduction of Dr. Black's offices and the display of his original manuscripts, published articles and books, his scientific instruments and many personal items of interest.

The building shown, while not intended as an exact replica, is correct in the shape and size of the rooms, the furnishings and their placement. The location was at Jacksonville, Illinois, and the period about 1895. Dr. Black practiced in these offices, which adjoined his residence, from about 1885 to 1897, when he moved to Chicago. The rooms were part of a two-story frame house and were entered from a hallway that occupied the position of the present porch. The back door opened into his combined dental laboratory, machine shop and chemical laboratory—what would be called today a research laboratory. The furnishings for the most part are the identical items used by Dr. Black. Replicas have been made of a few things which had been lost or destroyed. In examining these items, it should be remembered that Dr. Black discontinued practice when he moved to Chicago in 1897, and the furnishings have been stowed away in various places, so that they hardly present the same appearance as when in use. Except for a few necessary repairs, they have been left as they were.

The display of Dr. Black's writings and scientific instruments should serve to impress each visitor with the stupendous work accomplished by one man. There are hundreds of original manuscripts, covering a period of fifty years. It would require several years of a stenographer's time to copy all that Dr. Black wrote. A careful view of the titles will recall the fact that Dr. Black was a student in many fields aside from dentistry. There are articles on the Solitary Wasps, the Seventeen-Year Locusts, the Earth Worm, Tornadoes, Fossil Woods, Our Police System, the Use of Books, Typhoid Fever, Scarlet Fever, Bright's Disease, Mechanic Arts, Morality, the Microscope and Its Uses, the Theory of Sight, City Water Works, several Indian stories, many articles on chemical subjects, articles on travel, etc., etc. For fifty years it was a part of his routine to make written record of his thoughts and work, and thus he left a rich legacy.

The scientific instruments, only a part of which were shown on account of the limited space, are in themselves a wonderful exhibition of the work of this man. Each instrument was designed for the purpose of working out some new problem. Most of them were made by Dr. Black in his own workshop.

The many diplomas and numerous gifts convey to the mind something of the esteem in which Dr. Black was held by his professional brethren. If to these, the kind words spoken and written could be added, no man would dare harbor the thought that he had lived in vain.

Dr. Black was a musician of ability, he played the piano, violin, cello, piccolo, flute and cornet. Three of his own instruments, the violin, cello and piccolo, were on display.

One of Dr. Black's manuscripts is entitled "How to Rest." One wonders if he ever rested, yet it was his custom to spend a month or six weeks of each summer in the woods or in travel. Some of his fishing tackle, photographs of the boat he designed, and a few outing photographs were shown to remind us that he was a good sportsman.

It seems fitting in connection with this exhibit to show pictures of Dr. Black's family. He left a rich heritage in four children, of the same sterling qualities that made for his greatness; children, like their father, possessed of an unswerving conscientiousness. Nor is any honor that can be bestowed too great for Mrs. Black. Those intimate with the home life of Dr. Black are emphatic in avowing that his greatness, in a large degree, was due to the devotion and self-sacrifice of Mrs. Black.

The association is under obligation to the members of the Black family who have contributed from their treasures and keepsakes that this display might be as complete as possible.

This exhibit follows too closely upon the death of Dr. Black for many to find the intensity of interest that would be displayed twenty-five or fifty years hence. If this collection can be maintained intact, each succeeding year will magnify its importance.

Consider the interest in a like collection pertaining to Robert Wooffendale, the first regularly educated dentist to practice in America, coming here in 1768; or of a display relating to Josiah Flagg, showing something connected with his capture and life on board a man of war in 1812; or of some personal touches relating to that great pioneer, Chapin A. Harris. Our knowledge of these men is restricted to written history.

### LEADING EVENTS IN THE LIFE OF DR. BLACK

Degrees: D.D.S., Missouri Dental College, 1877; M.D., Chicago Medical College, 1884; Sc.D., Illinois College, 1892; LL.D., Northwestern University, 1898; Sc.D., University of Pennsylvania, 1915.

Born near Winchester, Scott County, Illinois, August 3, 1836.

Family moved to a farm seven miles southeast of Virginia, in Cass County, Illinois, in 1845.

Attended country school about three months each winter.

Studied medicine with Dr. Thomas G. Black, a brother, at Clayton, Illinois, 1853-1856.

Studied dentistry with Dr. J. C. Speer, Mt. Sterling, Illinois, 1857.

Practiced dentistry at Winchester, Illinois, 1858-1862.

Enlisted in 129th Illinois Volunteers, 1862.

In hospital at Louisville, Kentucky, six months, and discharged for disability, 1863.

Practiced dentistry in Jacksonville, Illinois, 1864-1897.

Joined Missouri State Dental Society, 1866.

Joined Illinois State Dental Society, 1868.

First important dental paper on "Gold Foil" read before Illinois State Dental Society, 1869.

President Illinois State Dental Society, 1870-1871.

Invented one of the first cord driven, foot power dental engines, 1870.

Lectured on pathology, histology and operative dentistry, Missouri Dental College, 1870-1880.

First president of the Illinois State Board of Dental Examiners, 1881-1887.

Wrote book, "The Formation of Poisons by Micro-organisms," 1884.

Professor of Dental Pathology, Chicago College of Dental Surgery, 1883-1889.

Introduced teaching of Dental Technics, Chicago College of Dental Surgery, 1887.

Wrote for the American System of Dentistry, chapters on "General Pathology," "Dental Caries," "Pathology of the Dental Pulp" and "Diseases of the Peridental Membrane," 1886.

Wrote book, "Periosteum and Peridental Membrane," 1887.

Voted life membership in Illinois State Dental Society, 1889.

Professor Dental Pathology and Bacteriology, Dental Department, University of Iowa, 1890-1891.

Wrote book, "Descriptive Anatomy of the Human Teeth," 1891.

Wrote series of articles entitled "The Management of Enamel Margins," Dental Cosmos, 1891.

Professor Dental Pathology and Bacteriology, Northwestern University Dental School, 1891-1897.

Chairman Section on Etiology, Pathology and Bacteriology, World's Columbian Dental Congress, 1893.

Report on Dental Nomenclature, World's Columbian Dental Congress, 1893.

Wrote series of articles entitled "An Investigation of the Physical Characters of the Human Teeth in Relation to Their Diseases and to Practical Dental Operations, Together with the Physical Characters of Filling Materials," Dental Cosmos, 1895-1896.

Dean and Professor of Operative Dentistry, Dental Pathology and Bacteriology, Northwestern University Dental School, 1897-1915.

President, National School of Dental Technics, 1897.

President, National Dental Association, 1900.

Awarded First Fellowship Medal by the Dental Society of the State of New York.

Special Guest at Annual Meeting of American Dental Society of Europe, 1906.

Wrote work on "Operative Dentistry," in two volumes, 1908.

Testimonial Banquet by the Chicago-Odontographic Society, 1910.

Awarded Miller Prize for most distinguished services for the advancement of dentistry, by the International Committee, Paris, 1910.

Wrote book on "Special Dental Pathology," 1915.

Died on his farm fifteen miles northeast of Jacksonville, Illinois, August 31, 1915.

### TRIBUTES TO DR. G. V. BLACK

He believed in his profession. Can anything more sad happen to a man than to live all his life in doing something in which he does not heartily believe? Dr. Black had no doubt that dentistry was worth while and worthy of him and his best efforts. So far as I knew him, he was not much concerned over the financial advantage his work rendered him, but he was profoundly convinced of the blessing that dentistry might confer upon humanity. He was a scholar uniquely fitted for the particular field in which he worked. He had imagination, without which creative scholarship is almost, if not quite, impossible; he had a keen, logical efficiency; he had boundless industry; and with all these qualities he had a consuming interest in his work. His liking for his profession, his courage and scholarship made him a great power, and, like Chaucer's priest, the way he pointed out to others he first went himself.—A. W. Harris, President, Northwestern University.

He had an insatiable desire for knowledge, he was a good listener, was open-minded, and seemed able to extract something of value from almost all with whom he came in contact. He could adapt himself to all classes of men, be they fishermen, logmen, or men of letters and refinement. Whether college or nature bred, if they had facts of practical value to present, they were sure to evoke his lively interest. He was an assiduous reader, he observed accurately, and he was a profound thinker. It may truly be said of him that his was a diversified mind, richly stored with knowledge of a multitude of subjects. On account of his accurate methods he rarely had to retrace his steps in his writings and discussions, but was ever ready to give considerate attention to any who showed a live interest in any line of human activity. My association with him for forty odd years has never caused me to doubt his worth as a man and friend.—Thomas L. Gilmer, D.D.S., *Digest*.

